# Characterization and Performance of Soy-Based Adhesives Cured with Epoxy Resin

**DOI:** 10.3390/polym9100514

**Published:** 2017-10-16

**Authors:** Nairong Chen, Peitao Zheng, Qinzhi Zeng, Qiaojia Lin, Jiuping Rao

**Affiliations:** College of Materials Engineering, Fujian Agriculture and Forestry University, Fuzhou 350002, China; fafuzpt@163.com (P.Z.); fjafuzqz@163.com (Q.Z.)

**Keywords:** gluability, water resistance, soy flour, thermal stability, biomass

## Abstract

Soy-based adhesives have attracted much attention recently because they are environmentally safe, low cost, and readily available. To improve the gluability and water resistance of soy-based adhesives, we prepared an enzyme-treated soy-based adhesive modified with an epoxy resin. We investigated the wet shear strength of plywood bonded with the modified adhesive using the boiling-water test. Fourier transformed infrared spectroscopy (FTIR) and ^1^H nuclear magnetic resonance analysis were used to characterize the reaction between epoxy groups and –NH_2_ groups in the modified soy-based adhesives. FTIR analysis confirmed the cross-linking structure in the cured adhesives. Viscosity and the solid content of soy-based adhesives gradually increased with the increasing amount of epoxy resin, but had little effect on its operability. Wet shear strength of plywood samples increased as the amount of epoxy resin was increased, whereas the inverse trend was observed regarding the water absorption of cured adhesives. Compared to an unmodified adhesive, the addition of 30% of epoxy resin increased the wet shear strength of plywood samples by 58.3% (0.95 MPa), meeting the requirement of the Chinese National Standard for exterior plywood. Differential scanning calorimetry and thermogravimetric analysis showed the improved thermostability of the cured adhesives after curing at 160 °C. These results suggest that epoxy resin could effectively improve the performance of enzyme-treated soy-based adhesives, which might provide a new option for the preparation of soy-based adhesives with high gluability and water resistance.

## 1. Introduction

There is growing interest in the development of sustainable materials based on biomass as a substitute to petroleum, mainly due to the non-renewability of fossil resources. The amount of petroleum-based adhesives consumed in the forestry industry is extensive. This has generated a strong desire to develop bio-based adhesives that can satisfy the needed properties, in addition to being eco-friendly under working conditions [1,2]. Bio-based proteins, such as soybean [3,4], cottonseed [5,6], and canola [7] products, are abundant renewable resources and have high activities. Bio-based adhesives derived from soybean are being widely studied because they are renewable and environmentally safe. However, applications of soy-based adhesives are limited due to their poor water resistance [8]. Various efforts, including chemical and enzyme treatment, have been attempted to overcome this problem and to improve gluability under wet conditions [9,10,11]. Most of these efforts are one-pot reactions where the modifiers are added in the preparation process of soy-based adhesives. These generally work well, but the resulting adhesives tend to have a short shelf-life. Therefore, blending the modifier as a cross-linker, or curing agent, before the application of soy-based adhesives is currently a popular option [12,13,14]. Resin appears to be an efficient and feasible curing agent for soy-based adhesives. A large number of reports have focused on resin-supported curing agents, such as polymeric methane diphenyl diisocyanate [15,16], melamine-formaldehyde resins [17,18], synthetic latex [19], and epoxide resins [20,21]. In particular, the epoxide resins have received special attention due to the fact that epoxy groups readily react with functional groups such as carboxyl, hydroxyl, and primary amine. In addition, most epoxide resins can be produced from the epichlorohydrin, which is considered a very promising sustainable resource [22]. Gu et al. [23] reported a soy-based adhesive system consisting of sodium hydroxide denatured soy flour and an epoxide compound (derived from ammonia and epichlorohydrin) was used in particle board and met the minimum industrial requirements of M-2 particleboard. Another soy-based adhesive with an epoxide compound and soy meal was used in plywood and met the type II wet shear strength and water resistance requirements for interior plywood [24]. Polyacrylamide and sodium dodecyl sulfate-denatured soy meal was mixed with epoxy resin, and a cross-linking network was formed during its curing process, improving the wet shear strength and water resistance [20]. Lei et al. [17] found that the mixing of epoxy resin and melamine-formaldehyde resin could be an efficient curing agent for soy-based adhesives with significantly improved gluability, and plywood bonded with the adhesives system could meet the requirement of exterior plywood. In addition, epoxide resins have also been used as the cross-linker of bio-based materials, such as starch or dextrin adhesives and fiber-reinforced composites, and such materials showed improved physicochemical properties [25,26,27]. Our previous work indicated that a soy-based adhesive showed improved resistance to water by treatment of defatted soy flour (DSF) prepared with a combination of enzyme, acid, salt, and alkali, due to the Maillard reaction between the hydrolyzed cell wall polysaccharides and the denatured soy proteins [28]. However, the low wet shear strength of the plywood bonded with this adhesive did not meet the requirement of interior plywood, so this approach to improve water resistance of the soy-based adhesives modified by epoxide resins has not been studied further.

The objective of our current study was to evaluate and characterize the soy-based adhesives, prepared from a combination of enzymatic and chemical reagents, and modified with epoxy resin. In particular, its use in plywood suitable for exterior applications was evaluated. The epoxy resin-modified soy-based adhesive was characterized by FTIR, ^1^H-NMR, differential scanning calorimetry (DSC), and thermogravimetric (TG) analysis, and then applied to the production of plywood samples for testing. The epoxy resin-modified adhesive system with improved thermal stability and gluability after curing is discussed.

## 2. Materials and Methods 

### 2.1. Materials

DSF (53.4% of crude protein, 36.3% of carbohydrate, and 7.5% of moisture) ground from defatted soy meal was obtained from Shandong Wonderful Industrial Group Co., Ltd. (Kenli, China). 98% of the DSF was passed through a 200-mesh screen. Hydrochloric acid, ferric chloride, and sodium hydroxide and other chemical reagents used in this study were analytical grade and purchased from Sinopharm Chemical Reagent Beijing Co., Ltd. (Beijing, China). Viscozyme^®^ L (from *Aspergillus aculeatus*) with 100 fungal beta-glucanase units (FBG) per gram (FBG/g) of enzyme activity was obtained from Novozymes (Bagsværd, Denmark). Commercial diglycidyl ether of bisphenol-A (DGEBA) epoxy resin (E-51, with epoxide number 0.50–0.54) was purchased from Sanmu Group (Wuxi, China). *Pinus massoniana* veneers (30 cm × 30 cm in size, 1.2 to 1.3 mm in thickness, and 10% to 12% of moisture contents) were supplied by Jianyang Luban Wood Industry Co., Ltd. (Jianyang, China).

### 2.2. Preparation of Soy-Based Adhesive

Soy-based adhesive preparation was made according to our previous work, with minor modifications [3,28]; forty grams of DSF were dissolved in 160 mL of distilled water in a three-necked flask and stirred for 40 min in a 35 °C water bath. The DSF slurry was then adjusted with hydrochloric acid solution to pH 5.1. After that, 50 FBG units of Viscozyme^®^ L was added to the DSF slurry, and incubated at 54 °C for 20 min. The enzyme-treated DSF slurry was then adjusted to pH 1.1 with a hydrochloric acid solution containing 0.5% (mass fraction) of ferric chloride, and then stirred for 33.0 min at 29 °C. Next, the slurry was adjusted to pH 11 with sodium hydroxide solution (30%) and cooled to room temperature. Finally, various mass fractions of epoxy resin (0%, 10%, 20%, 30%, 40% or 50% of the DSF mass) were added to the slurry and stirred continuously for 10 min to obtain the epoxy resin-modified soy-based adhesives used in the study.

### 2.3. Characterization

**FTIR analysis.** The analysis was carried out using a Nicolet 380 FTIR spectrometer (Thermo Fisher Scientific, Waltham, MA, USA) at a resolution of 4 cm^−1^ for 64 scans in the spectral range of 500–4000 cm^−1^. Each soy-based adhesive sample was ground into a powder and 2 mg was mixed with KBr (200 mg) to form pellets (20 MPa for 3 min) for analysis. Nicolet OMNIC software (Version 8.2, Thermo Fisher Scientific, Waltham, MA, USA) was used for instrument control and spectral data analysis. The spectrum was baseline corrected and normalized to the CH_2_ and CH_3_ stretching vibration region (2960–2820 cm^−1^) [29,30].

**^1^H-NMR analysis.** A sample of soy-based adhesive powder (10 mg) was dissolved into 0.5 mL of D_2_O, filtered, and the resulting solution was placed in a 5-mm NMR tube for ^1^H-NMR analysis. The spectra of sample were recorded on a Bruker AVANCE III 500 spectrometer (Fällanden, Switzerland) equipped with a 5 mm cryoprobe and operating at a ^1^H frequency of 500 MHz and constant temperature of 298 K. A total of 256 free induction decays (FIDs) were collected with 64,000 data points over a spectral width of 16 ppm. One-dimensional pulse-acquired spectra were recorded using a pulse width of 3.4 μs with a 2.0 s water pre-saturation delay. Spectral phase and baseline corrections were performed using MestReNova software (Mestrelab Research S. L., Version: 6.1.0–6224, A Coruña, Spain).

**DSC and TG analysis.** The thermal stabilities of the bio-based adhesive samples were determined by a NETZSCH STA449F3 TGA instrument (NETZSCH Co., Selb, Germany). Samples (5.0 mg in a 70 µL alumina pan) were heated from 25 to 300 °C at a rate of 10 °C/min under nitrogen gas (30 mL/min). 

Two types of adhesive samples were used in FTIR, ^1^H-NMR, DSC, and TG analysis: (i) uncured adhesive samples were first prepared at −48 °C and 6.5 Pa dry vacuum for 48 h; and (ii) cured adhesive samples were carefully collected from the edges of plywood. 

**Viscosity.** A Brookfield DV-III viscometer (USA) was employed to inspect the viscosity of soy-based adhesives. The test was performed at 25 ± 1 °C and 30 rpm using spindle number 4 and expressed in Pa·s. The viscosity was measured in triplicate.

**Solid content.** Soy-based adhesive (4.0 g, *m*_1_) was weighed into a weighing bottle. The bottle was then opened, and the sample was dried in an oven at 120 °C until it reached a constant mass (*m*_2_). The solid content of the bio-based adhesives was calculated using the following Equation (1):

Solid content = *m*_2_/*m*_1_ × 100% (1)

The results are presented as the mean of three replicates.

**Hydrophilicity.** Water absorption of the cured adhesives in the distilled water were studied according to a literature procedure [31]. Each sample (1.000 g, *m*_1_) was submerged in 20 mL distilled water for 24 h at 25 ± 1 °C. After treatment, the extra water on the surface of the samples was removed by a paper towel, and weighed again (*m*_2_). The water absorption of the cured adhesives was calculated as shown in Equation (2):

Water absorption = (*m*_2_ − *m*_1_)/*m*_1_ × 100% (2)

The results are presented as the mean of three replicates.

**Figure 1:**
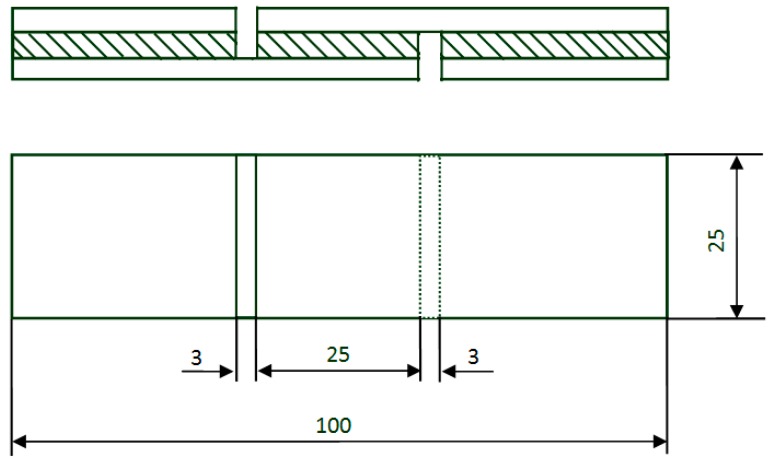
The diagram of shear strength test specimen (units: mm).

**Gluability.** Soy-based adhesives were applied to three-ply wood by brushing about 140 g/m^2^ of the adhesive on each *Pinus massoniana* veneer. The assembly time, pressing temperature, pressure, and time were 12 min, 160 °C, 1.0 MPa, and 3.6 min, respectively. After storing in a chamber at 25 °C and 50% RH (Relative humidity) for 24 h, each piece of plywood was cut into 10 samples (100 mm × 25 mm, Figure 1). The gluabilities of the developed soy-based adhesives were evaluated by the glue line wet shear strength of plywood samples subjected to a boiling-water soaking pretreatment. In the boiling-water soaking pretreatment, the plywood specimens were soaked in boiling water for 3 h and then cooled to room temperature for 10 min. A tensile testing machine (MTS, Shenzhen, China) with a crosshead speed of 10 mm/min was used to test the wet shear strength [24,32]. For each adhesive treatment, the three-ply plywood was prepared in triplicate. The number of test specimens for each combination was 30, and the average wet shear strength was calculated from their results. Microsoft Office Excel 2007 (Redmond, WA, USA) was the software used for data analysis (Tukey test).

## 3. Results and Discussion

### 3.1. FTIR Analysis

**Figure 2:**
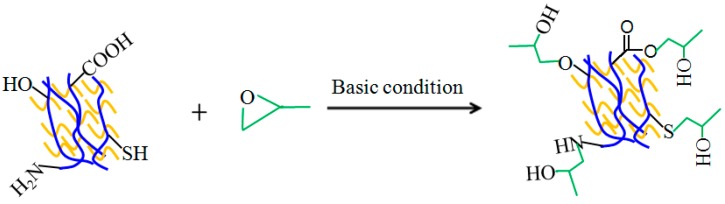
Possible reaction between functional groups in soy-based adhesives and the epoxide group of epoxy resin.

The DSF slurry treated with a combination of enzyme, acid, salt, and alkali can improve reactive functional groups, such as –NH_2_, –COOH, –SH, and –OH, which can then readily react with the epoxy groups at high temperature [20,33]. A possible reaction pathway is shown schematically in Figure 2. 

In order to verify the cross-linking reaction of cured soy-based adhesives, FTIR spectra (Figure 2) of the soy-based adhesives with and without epoxy resin (50% of the DSF mass) were performed. 

**Figure 3:**
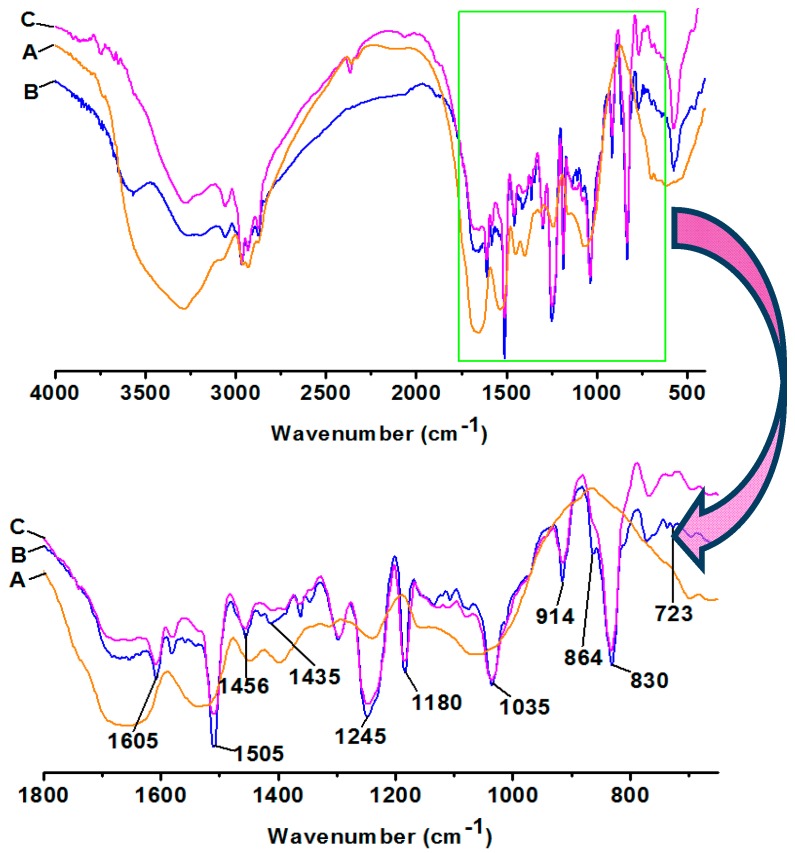
FTIR spectra of soy-based adhesives (A: soy-based adhesives were prepared from DSF via the treatment of Viscozyme L in combination with acid, salt, and alkali; B: epoxy resin-modified soy-based adhesives were prepared from DSF via treatment of Viscozyme L in combination with acid, salt, and alkali (before curing); and C: sample B after curing).

Figure 3 shows that the bands at 720, 1430–1470 cm^−1^ (aliphatic hydrocarbon) [3,34], and 830, 1180, 1505, 1605, 2850–2960 cm^−1^ (carbon in aromatic nucleus) [35], 915 cm^−1^ (epoxy groups) [34,35], 1035 cm^−1^ (aliphatic ether) [3], and 1230–1250 cm^−1^ (aromatic ether) [24,36], which, due to epoxy resin absorbance, were not detected in the sample A, but were present in samples B and C, suggesting the existence of epoxy resin in the cured soy-based adhesives. This implied that the additive amount of epoxy resin can be less than 50% of the DSF mass when curing the soy-based adhesives. The peaks at 3500 and 1640 cm^−1^ with sample B were assigned to N–H bending and C–N stretching vibration of soy protein, respectively, and were weaker than on sample A. It is possible that this is because the enzyme treatment of DSF increases the functional groups (e.g., –NH_2_) in soy-based adhesives, leading to the chemical reaction that occurred after the epoxy resin and soy-based adhesive is blended. Therefore, it is better to separate the soy-based adhesives and epoxy resin in storage, and blend them together during the procedure. Compared to the spectrum of sample B, sample C showed weaker peaks at the hydroxyl and amide groups (3300–3600 cm^−1^) and epoxy groups (914 cm^−1^), and had a slight intensity increase at the peak of the carbonyl group in ester linkages (1740 cm^−1^) which can be attributed to the cross-linking reactions shown in Figure 2 [20,31]. These results indicate that the chemical reaction between these groups was the key reaction in the curing process of the epoxy resin-modified soy-based adhesives. The decreased hydrophilic groups (e.g., –NH–, –OH, etc.) and increased cross-linkage in cured epoxy resin-modified soy-based adhesive will more likely improve its water resistance [37].

### 3.2. ^1^H-NMR Analysis

**Figure 4:**
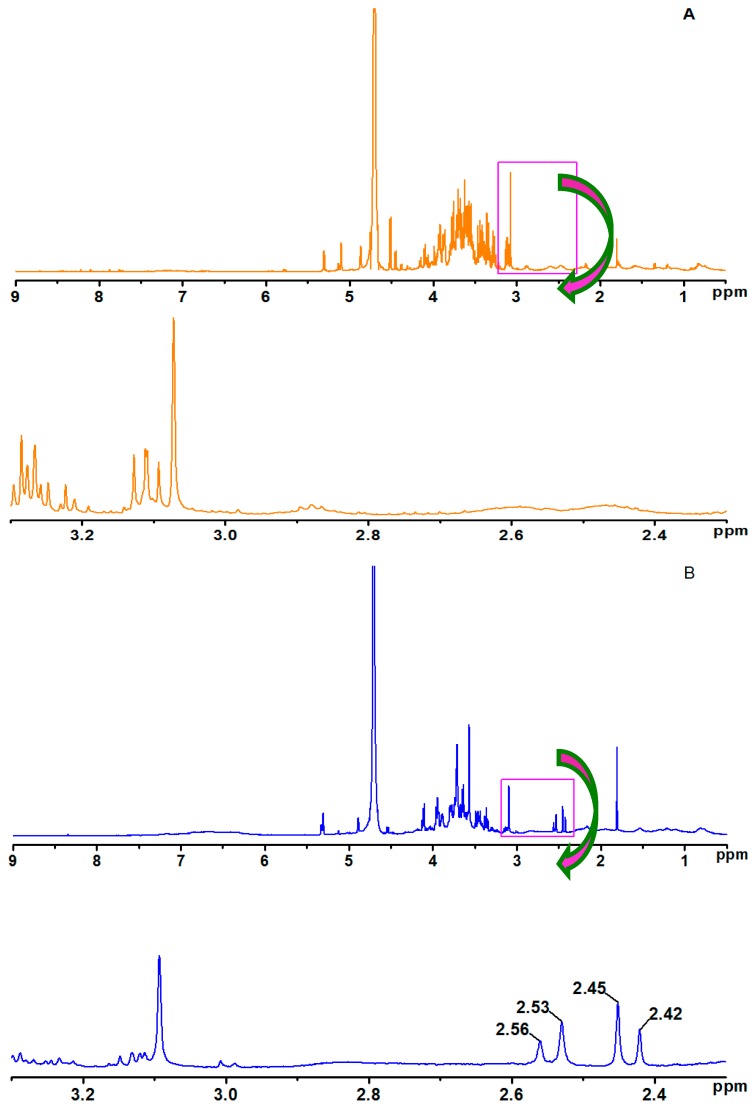
^1^H-NMR spectra of soy-based adhesives ((**A**) soy-based adhesives were prepared from DSF via the treatment of Viscozyme L in combination with acid, salt, and alkali; (**B**) epoxy resin-modified soy-based adhesives were prepared from DSF via treatment of Viscozyme L in combination with acid, salt, and alkali (before curing)).

To further confirm the chemical reaction after epoxy resin was blended with soy-based adhesives, ^1^H-NMR analysis was performed. As shown in Figure 4, the spectra of sample B (Figure 4B) displayed the chemical shifts for methyl (2.40–2.60 ppm) on the bisphenol A of epoxy resin, but none of these chemical shifts were found in the spectra of sample A (Figure 4A). In addition, the chemical shifts for methylene (2.34 and 2.70 ppm) and methyne (2.80 ppm) on the epoxy group structure of epoxy resin were not seen in either samples. These results suggest that the epoxy resin structure was introduced to the soy-based adhesive because the epoxy resin is undissolved in D_2_O [38,39]. This implied that the components of soy-based adhesive can react with the epoxy resin during the mixing process, which is also in agreement with the analysis of FTIR.

### 3.3. Viscosity and Solid Content

The viscosity of adhesives should be low enough to give good spreading and penetration. Viscosity also constrains the solid content of adhesives because viscosity increases with solid content [40]. Therefore, the effect of viscosity and solid content on soy-based adhesives’ operability is very important in determining proper additive amounts of epoxy resin. Table 1 shows that the viscosity and solid content of soy-based adhesives gradually increased with the additive amount of epoxy resin (*p* < 0.05). Ten percent of epoxy resin increased the viscosity and solid content of soy-based adhesives by 210.4% and 8.8%, respectively, and further increased to 249.4% and 34.5% when 50% of epoxy resin was loaded, suggesting that the 10–50% of the additive amount of epoxy resin has no significant effects on the operability of soy-based adhesives (*p* > 0.05). The change in viscosity can be explained by the increased solid content and/or molecular weight of soy-based adhesives. Furthermore, the slightly increased viscosity and solid content of soy-based adhesives will be good for the gluability on wood surfaces due to avoidance of over-penetration to form a starved bond line [2,41].

### 3.4. Hydrophilicity and Gluability

Hydrophilicity and gluability both impact the possible applications of soy-based adhesives. Both can be evaluated from *Pinus massoniana* plywood bonded with epoxy resin-cured soy-based adhesives after a boiling-water soaking treatment, while the hydrophilicity of the cured soy-based adhesives can be evaluated by a water absorption test as well. As shown in Table 1, the wet shear strength of all of the samples increased as the additive amount of epoxy resin was increased (*p* < 0.05), whereas the inverse tendency was found in the water absorption of cured soy-based adhesives (*p* < 0.05), indicating that the water-resistance of soy-based adhesives was improved by epoxy resin because the hydrophilic groups were consumed by epoxy groups, resulting in the increased cross-linkage structure. This was also in agreement with the FTIR analysis and previous literature [17,42]. When 10% of epoxy resin was added, the water absorption decreased by 40.0% (*p* < 0.05), which is a significant difference of a 6.7% improvement of the wet shear strength (*p* > 0.05). It is possible that this is because the treatment temperature of plywood was 100 °C, and for the water absorption test it was only 23 °C. After 30% of epoxy resin was added, the wet shear strength of the samples increased by 58.3% (0.95 MPa), which met the requirement of the Chinese National Standard GB/T 9846-2004 for exterior plywood (*Pinus massoniana* plywood ≥ 0.89 MPa) [28]. The wet shear strength increased sharply to 1.31 MPa at 50% additive amount of epoxy resin, which was significantly higher than our previous studies [3]. This could be ascribed to formation of the better cross-linking structures in cured soy-based adhesives because the increased functional groups after enzymatic treatment of DSF is also readily reacted with the epoxy group during the curing process [8]. 

### 3.5. DSC and TG Analysis 

**Figure 5:**
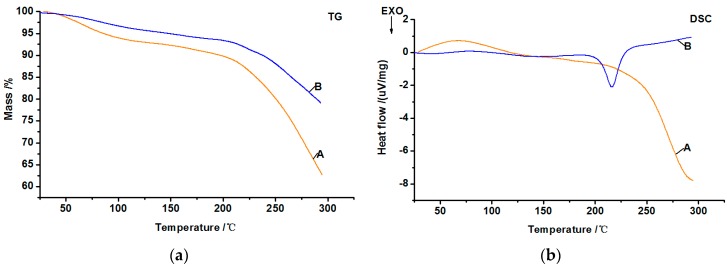
TG (**a**) and DSC (**b**) curves of soy-based adhesives (A: soy-based adhesives were prepared from DSF via treatment of Viscozyme L in combination with acid, salt, and alkali; B: epoxy resin-modified soy-based adhesives were prepared from DSF via treatment of Viscozyme L in combination with acid, salt, and alkali).

To further investigate the curing process of the epoxy resin-modified soy-based adhesives, DSC and TG analyses were performed (Figure 5). The differences in hydrophilicity and curing status of adhesive samples are reflected by its mass loss at 100 °C (water boiling point) and 160 °C (hot-pressing temperature of the plywood), respectively. The change of mass loss and heat flow under 160 °C are displayed in Table 2.

The results in Figure 5 and Table 2 show that the samples decreased in mass with an increase in temperature, but the change in heat flow depends on different temperature stages. The endothermic peak at under 100 °C could be found in DSC analysis, which was caused by volatilization of low boiling point components (e.g., residual of water) after the absorption of heat [43]. The mass loss of sample A was higher than that of sample B at 100 °C, and the endothermic peak area and endothermic end-point temperature of sample A was lower than that of sample B, but the difference in the endothermic peak temperature was only 2.8 °C, suggesting that the decreased hydrophilicity of soy-based adhesives occurred after mixture with the epoxy resin. This may be attributed to the hydrophobic property of the epoxy resin and/or the hydrophilic functional groups (e.g., –NH_2_) in the soy-based adhesives’ reaction with the epoxy group [20,44]. The mass loss of samples A and B increased by 34.60% and 63.96%, respectively, when the temperature was raised to 160 °C. An exothermic peak was observed at 137.3 °C in sample B, but was not obvious in sample A, indicating that a condensation reaction might have occurred in the heating process of sample B [45]. Therefore, the curing temperature of this adhesive-based composite should not be lower than 137.3 °C, and the temperature of 160 °C was sufficient for prepared plywood. 

The mass loss of sample A and B sharply increased when the temperature was higher than 215 °C, implying that decomposition might occur in the cured soy-based adhesives. The exothermic peak that appeared in sample B at 215 °C could be explained by the residual epoxy resin in the sample B reaction with the secondary amine structure of soy-based adhesives. However, the mass loss and heat flow of sample B was significantly lower than sample A when the temperature was higher than 230 °C, which indicated the better thermo-stability of sample B after curing, because the three-dimensional structure was formed during the curing process. These results also verified the gluability analysis of the epoxy resin-modified soy-based adhesives. 

## 4. Conclusions

In this work, we showed that epoxy resin could enhance the properties of a soy-based adhesive prepared with a combination of Viscozyme L, acid, salt, and alkali-treated DSF. Epoxy resin-modified soy-based adhesives had significantly improved gluability, water resistance, solid content, viscosity, and thermal stability. However, there should be separation of epoxy resin and soy-based adhesives in storage because epoxy groups readily reacted with –NH_2_ groups. Wet shear strength of *Pinus massoniana* plywood bonded with 30% of DSF mass epoxy resin-modified soy-based adhesives could meet the requirement of the Chinese National Standard for exterior plywood. Compared to current modifications of soy-based adhesives, our study provides a new option for preparation of soy-based adhesives with high gluability and water resistance.

## Figures and Tables

**Table 1:** The properties of epoxy resin-modified soy-based adhesives.

The Amounts of Epoxy Resin (%)	Viscosity ± SD (Pa·s)	Solid Contents ± SD (%)	Water Absorption ± SD (%)	Wet Shear Strength ± SD (MPa)
0	0.816 (± 0.021) ^a^	19.4 (± 0.21) ^a^	6.52 (± 0.11) ^a^	0.60 (± 0.15) ^a^
10	2.533 (± 0.011) ^b^	21.1 (± 0.13) ^ab^	3.91 (± 0.08) ^b^	0.64 (± 0.12) ^a^
20	2.576 (± 0.017) ^b^	22.3 (± 0.18) ^ac^	3.65 (± 0.10) ^b^	0.83 (± 0.21) ^ab^
30	2.668 (± 0.014) ^b^	23.6 (± 0.24) ^ac^	3.64 (± 0.04) ^b^	0.95 (± 0.17) ^ac^
40	2.719 (± 0.013) ^b^	24.7 (± 0.16) ^bc^	3.56 (± 0.05) ^b^	1.15 (± 0.07) ^bc^
50	2.851 (± 0.009) ^b^	26.1 (± 0.19) ^c^	3.54 (± 0.07) ^b^	1.31 (± 0.13) ^c^

SD represents standard deviation (*n* = 3). Values followed by the same superscript letter (e.g., a, b or c) in same column are not significantly different (*p* > 0.05).

**Table 2:** Weight loss and heat flow change in soy-based adhesives under 160 °C.

Entry	Mass Loss at 100 °C (%)	Mass Loss at 160 °C (%)	Endothermic Peak Temperature (°C)	Endothermic Peak Area (uV·s/mg)	Endothermic End-Point Temperature (°C)
A	6.04	8.13	68.1	162.6	117.1
B	3.33	5.46	70.9	30.3	97.8
